# Oral antigen exposure in extreme early life in lambs influences the magnitude of the immune response which can be generated in later life

**DOI:** 10.1186/1746-6148-9-160

**Published:** 2013-08-12

**Authors:** Rachelle M Buchanan, Sonja Mertins, Heather L Wilson

**Affiliations:** 1 Vaccine and Infectious Disease Organization (VIDO), University of Saskatchewan, Saskatoon S7N 5E3, SK, Canada

**Keywords:** Lambs, Neonate, Ovalbumin, Mucosal, Oral

## Abstract

**Background:**

Previous investigations in newborn lambs determined that adenovirus-mediated expression of antigen to a localized region of the gut induced antigen-specific mucosal and systemic immunity. These experiments were limited in that the localized region of the gut to which antigen was introduced was sterile and the influence of colostrum on the antigen was not assessed but they do suggest that mucosal vaccines may be an effective vaccination strategy to protect neonatal lambs. We propose that persistent oral antigen exposure introduced in extreme early life can induce immunity in lambs, despite the presence of commensal bacteria and colostrum.

**Results:**

To test this hypothesis, conventionally raised newborn lambs (n = 4 per group) were gavaged with ovalbumin (OVA) starting the day after birth for either a single day (2.27 g), every day for 3 days (0.23 g/day), or every day for 3 days then every second day until nine days of age (0.023 g/day). Lambs gavaged with OVA for 3 to 9 days developed significant serum anti-OVA IgG titres (p < 0.05), but not IgA titres, relative to control lambs (n = 4) after 3 and 4 weeks. At 4 weeks of age, lambs were immunized with OVA in Incomplete Freund’s Adjuvant via intraperitoneal (i.p.) injection then lambs were euthanized at 7 weeks. Serum anti-OVA IgG titres were further augmented after i.p. immunization indicating immunity persisted and tolerance was not induced. Serum IgA titres remained low regardless of treatment. It is known that i.p. priming of sheep with antigen in Freund’s complete adjuvant leads to an enhanced number of IgA and IgG antibody containing cells in the respiratory mucosa (Immunology 53(2):375–384, 1984). Lambs gavaged with a single bolus of 2.27 g OVA prior to i.p. immunization showed very low titres of anti-OVA IgA in the lung lavage. These data suggest that a single, high dose exposure to OVA can promote tolerance which impacts response to systemic vaccination in later life. Lambs gavaged with 0.023 g OVA for 9 days (Group C) generated significant anti-OVA IgA titres in lung (p < 0.001) compared to negative control lambs but no additive effect was observed compared to parenteral control lambs. When splenocytes were re-stimulated with OVA *ex vivo*, all groups failed to show increased lymphocyte proliferation or interferon (IFN)-γ production relative to the parenteral control group.

**Conclusions:**

In agreement with our hypothesis, persistent low dose antigen exposure primes humoral antibody production in serum in conventionally raised newborn lambs. In contrast, a single high dose bolus of antigen triggered oral tolerance which negatively impacted the quality and magnitude of the immune response to i.p. immunization in later life. These tangential responses are important as they indicate that the dose and/or repeated oral exposure to antigen, such as that which may be found in the neonate’s environment, may promote immunity or alternatively it may negatively impact responses to parenteral vaccination.

## Background

In the neonatal period, lambs are highly susceptible to infectious disease [1]. Maternal antibodies from colostrum protect lambs from diseases but passive immunity begins to wane markedly in the first few months after birth, leaving the neonate susceptible to disease [2]. Successful neonatal vaccination would be an efficacious and cost-effective way to protect lambs from disease during the perinatal periods but this is largely not practised due to the limited success reported for vaccination using parenteral vaccination methods [3-5]. New approaches to effectively vaccinate newborns so they produce their own immune responses when passive immunity wanes are highly sought.

Oral tolerance is a suppressive mechanism designed to prevent the host immune system from overreacting to innocuous antigens [6]. Once oral tolerance is induced, it is sufficiently robust that subsequent exposure to that antigen, even via a systemic route, suppresses immunity [7,8].

Previously, it was assumed that the neonatal immune system was too immature to mount an effective immune response to vaccines [9,10]. Numerous studies in ruminants have confirmed that fetal lambs can mount an effective immune response to antigens [11-14] Despite success in fetal lambs, several reports showed that newborn ruminants respond poorly to vaccination, possibly due to interference from passively acquired maternal antibodies [3-5]. In contrast, others report that, depending on the dose of the antigen, the type of adjuvant used, and the type of antigen-presenting cell targeted, neonates can mount an effective immune responses [15]. Mutwiri et al. (2001) determined that the gut-associated lymphoid tissues (GALT) of newborn lambs was immune competent [15]. Further, they used an intestinal ‘loop’ model to facilitate the localization of human adenovirus coding for TgD, a bovine herpes virus antigen, to a single jejunal Peyer’s Patch (PP) [15]. They suggested that the adenovirus would produce a consistent supply of TgD to the jejunal PP and, indeed, the majority of enterically-immunized lambs responded with strong mucosal and systemic immunity [15]. However, they acknowledge that the intestinal ‘loops’ were not in direct contact with colostrum which may contain antibodies or non-specific inhibitors that could potentially inactivate the adenovirus vector or otherwise interfere with a vaccine. We hypothesized than if neonatal lambs responded with immunity after enteric immunization with a consistent dose of antigen (delivered by adenovirus), they should respond to persistent oral antigen immunization even in the presence of commensal bacteria and colostrum. To test this hypothesis, conventionally reared neonatal lambs were gavaged with 2.27 g – 0.023 g ovalbumin (as an experimental antigen) as a single bolus or up to 6 times over a 9 day period starting the day after birth. Systemic and mucosal immune responses were assessed using a variety of assays to determine humoral and cell-mediated immunity.

## Results

### Serum anti-OVA IgG titres induced in newborn lambs in response to oral gavage is sensitive to dose and persistence of antigen exposure

**Figure 1:**
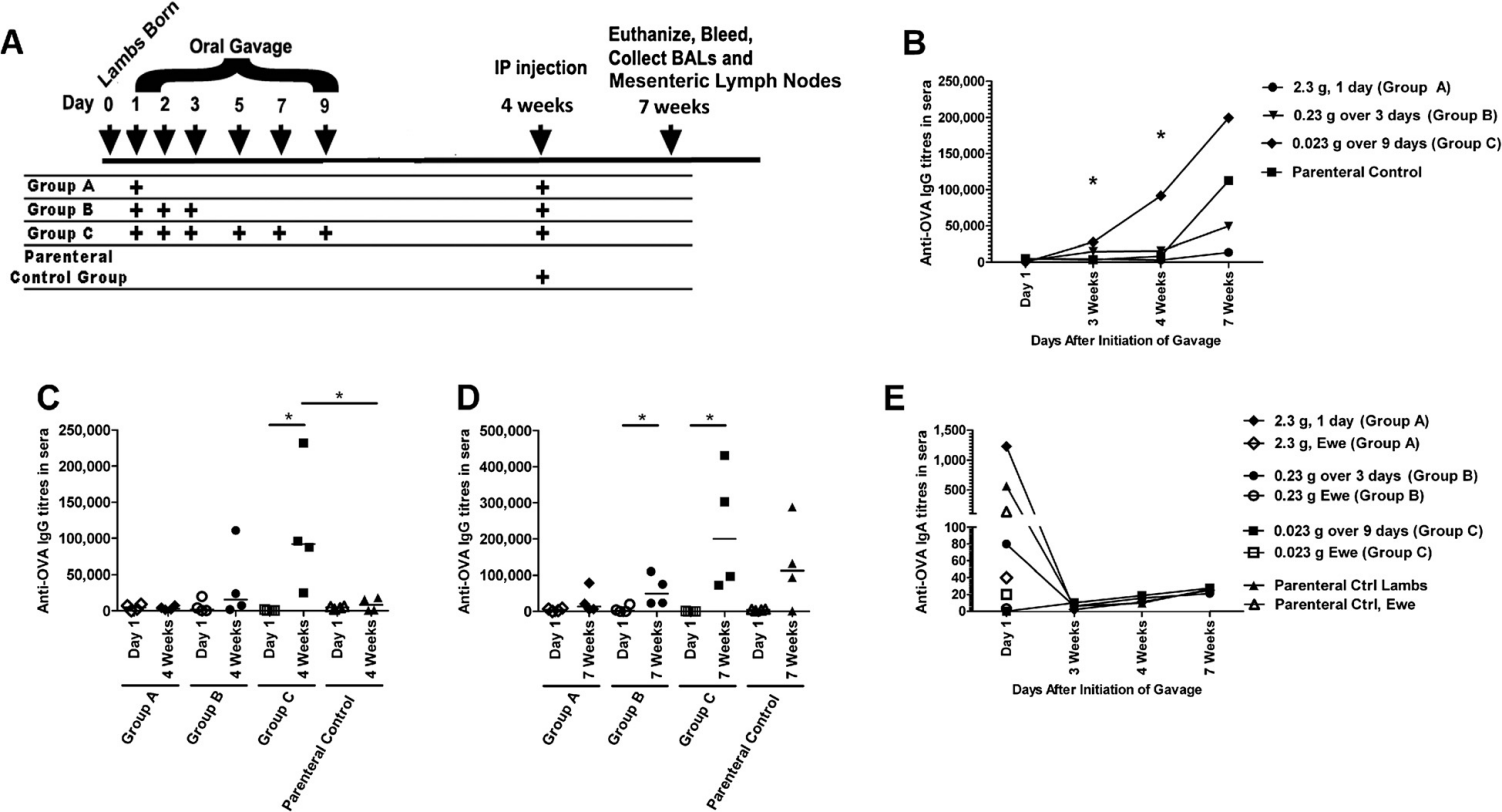
**OVA-specific humoral immune responses in serum from newborn lambs gavaged with OVA then i.p. immunized with OVA at 4 weeks of age. (A)** Lambs (n = 4/group) were gavaged with OVA at day 1 (2.27 g OVA; Group **A)**, on day 1, 2 and 3 after birth (0.23 g OVA/day; Group **B)** and for the first 3 consecutive days after birth as well as day 5, day 7 and day 9 (0.023 g OVA/day; Group **C)**. All groups including the parenteral control group were i.p. immunized at 4 weeks of age with OVA in IFA. At 7 weeks of age, lambs were sacrificed, and spleens and lung lavages were harvested. Blood was obtained after 3 weeks, 4 weeks and at time of death. Kinetics of the anti-OVA serum IgG production are shown in **(B)**. OVA-specific serum IgG titres were measured 4 weeks **(C)** after birth, as well as 3 weeks post i.p. immunization **(D)**. Kinetics of the anti-OVA serum IgA production are shown in **(E)**. In **(C** and **D)** each data point represents an individual animal and median values are indicated by horizontal lines. In **(B** and** E)**, data are represented as median from each group. *p < 0.05.

Lambs were gavaged with OVA at day 1 (2.27 g OVA; Group A), on day 1, 2 and 3 after birth (0.23 g OVA/day; Group B) and for the first 3 consecutive days after birth as well as day 5, day 7 and day 9 (0.023 g OVA/day; Group C) as detailed in Figure 1A. Lambs were bled on their day of birth and at 4 weeks and 7 weeks of age. Lambs had unrestricted access to maternal colostrum and were colonized by commensal bacteria. All lambs were i.p.-injected with 10 mg OVA plus Incomplete Freund’s Adjuvant (IFA) at 4 weeks of age, and euthanized at 7 weeks of age. Bronchoalveolar lavages (BALs) and spleens were collected at time of death. We used enzyme-linked immunosorbant assays (ELISAs) to evaluate anti-OVA IgG and IgA tires in blood sera and respiratory mucosa. The overall kinetics of the OVA-specific immune response as measured by serum IgG are depicted in Figure 1B and the data shows that there was a significant difference in serum anti-OVA IgG titres between the groups at 4 weeks of age. When the IgG titres were compared for each group relative to their serum titres at day 1, we observed that after 4 weeks there was a significant induction of anti-OVA IgG in the lambs gavaged for up to 9 days with 0.023 g OVA (Group C; p < 0.05, Figure 1C). Further, this group of lambs (Group C) showed significant induction of anti-OVA IgG titres after 4 weeks (p < 0.05, Figure 1C) relative to the parenteral control group (note this group had only received saline up to this point and can be regarded as a negative control group.) These data indicate that despite being conventionally reared (i.e. with normal commensal flora and with access to colostrum), lambs orally vaccinated for 9 days with 0.023 g OVA alone showed significant induction of anti-OVA IgG in serum. Oral administration of 2.27 g OVA the day after birth (Group A) or 0.23 g OVA daily for 3 days after birth (Group B) did not promote significant induction of serum anti-OVA IgG titres which suggest that whether lambs respond to oral antigen with immunity or not is dependent upon dose or persistence of exposure.

After 7 weeks (which was 3 weeks after i.p. immunization), there was a trend towards increased anti-OVA IgG in all groups, with Group C and the parenteral control group showing the highest median values (Figure 1D). The group of lambs gavaged for 9 days with 0.023 g OVA (Group C) had approximately 2 fold higher anti-OVA IgG titres after 7 weeks (Figure 1D) relative to the titres observed at 4 weeks of age (Figure 1C). Because it was clear from Figure 1C that oral exposure of lambs from Group C showed significant induction of serum anti-OVA IgG, this further 2 fold increase in serum IgG after 7 weeks indicates that even after re-exposure to OVA by a systemic route, immunity, not tolerance, persisted. Lambs gavaged for 3 days (Group B) showed significant induction of anti-OVA IgG relative to titres from day 1 (p < 0.05, Figure 1D) but because lambs were i.p.-immunized at 4 weeks of age, it is not clear if serum anti-OVA IgG titres quantified after 7 weeks indicate induction of mucosal immunity from oral OVA exposure or systemic immunity from i.p. administered OVA. Lambs gavage with a single bolus of 2.27 g OVA (Group A) showed a trend towards decreased antibody production relative to the parenteral control group after 7 weeks but the difference was not statistically significant (Figure 1D). These data suggest that a single high dose bolus of antigen may promote immune tolerance. Together these results indicate that 9 day oral exposure to low doses of antigen triggers serum humoral immunity, not oral tolerance. In contrast, a single, high dose exposure may induce oral tolerance which negatively impacts parenteral immunization.

When we assessed the anti-OVA IgA titres in serum, we observed that some lambs across all groups showed robust anti-OVA IgA in serum at (Day 0). When we investigated the ewe serum for anti-OVA IgA, it was present at high levels in the ewes whose lambs showed high anti-OVA IgA titres (Figure 1E). Regardless of whether the lambs consumed high or low anti-OVA IgA titres, all lambs failed to produce significant anti-OVA IgA serum titres over time (Figure 1E).

### Anti-OVA antibodies in respiratory mucosa induced in newborn lambs in response to oral gavage is sensitive to dose and persistence of antigen exposure

**Figure 2:**
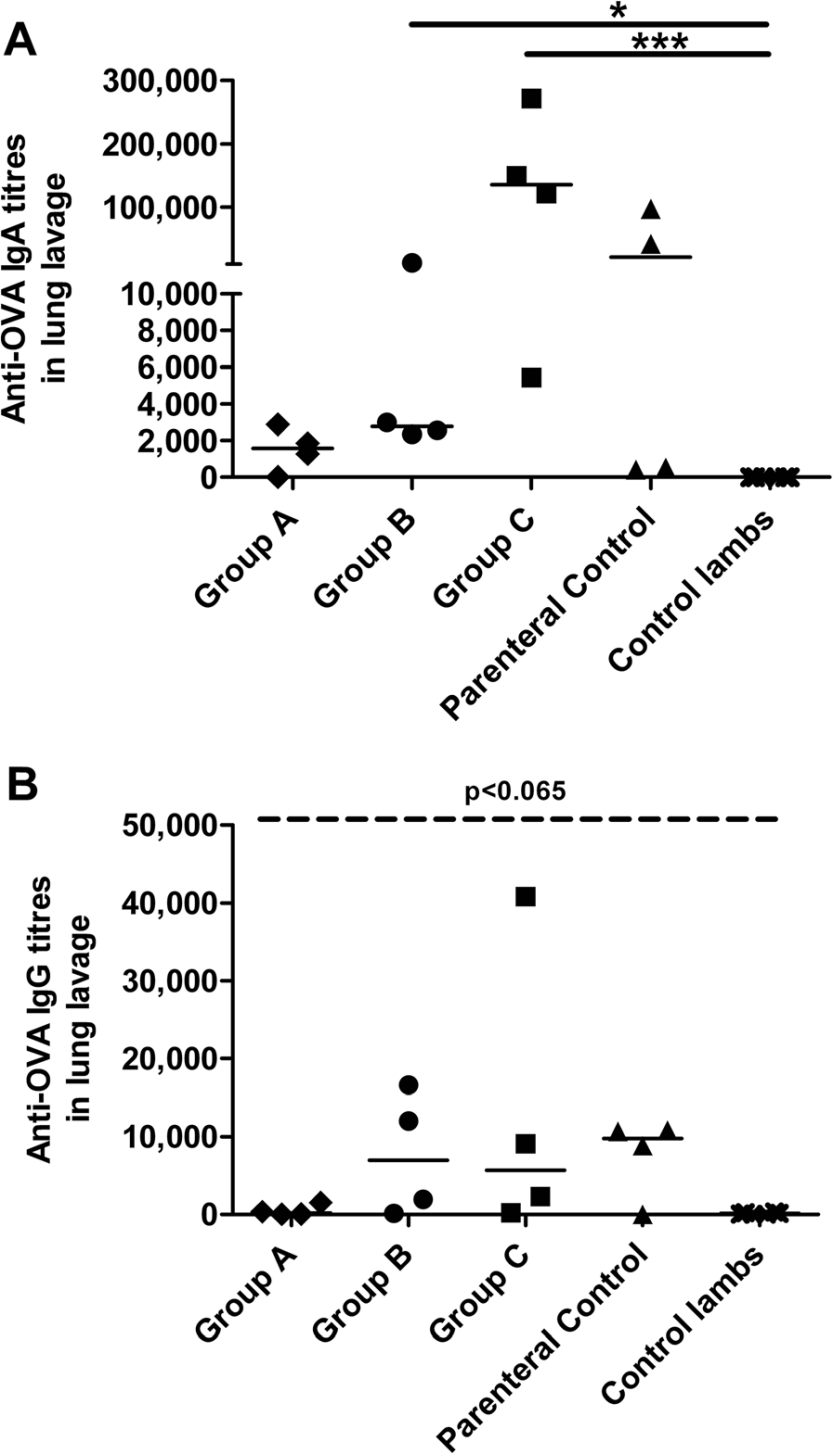
**OVA-specific humoral immune responses in lung washes from newborn lambs gavaged with OVA then i.p. immunized with OVA at 4 weeks of age.** Lambs (n = 4/group) were gavaged and i.p. immunized as described in Figure 1A. Control newborn lambs were not gavaged or immunized with OVA. Lung lavages were collected 3 weeks post i.p. immunization and OVA-specific serum IgA **(A)** and IgG titres **(B)** were measured. ELISA titres are expressed as the reciprocal of the highest dilution resulting in a reading of two standard deviations above the negative control. Each data point represents an individual animal and median values are indicated by horizontal lines. *p < 0.05, ***p < 0.001.

According to the ‘Common Mucosal Immune System’ theory, antigen-sensitized precursor B and T lymphocytes generated at one mucosal site (i.e. such as the gut) can be detected at anatomically remote and functionally distinct compartments (such as the respiratory mucosa) [16-21]. It is known that i.p. priming of sheep with antigen in Freund’s complete adjuvant leads to an enhanced number of IgA and IgG antibody containing cells in the respiratory mucosa [22]. We predicted that neonatal lambs injected with OVA in IFA by the i.p. route alone would result in significant IgA antibodies in the bronchoalveolar lavage. We measured anti-OVA antibody titres in lung washes 3 weeks post i.p. immunization. Results indicated that 2 of the 4 animals failed to produce anti-OVA IgA in the respiratory mucosa but the remaining two animals produced strong anti-OVA IgA titres (Figure 2A). We wished to establish whether oral gavage of newborn lambs influenced the mucosal anti-OVA IgA titres produced by i.p. immunization alone. Lambs gavaged with a single bolus of 2.27 g OVA prior to i.p. immunization showed very low titres of anti-OVA IgA in the lung lavage which may indicate induction of oral tolerance, although it did not meet the criteria of statistical significance relative to the parenteral control group. Lambs gavaged with 0.023 g OVA for 9 days (Group C) generated significant anti-OVA IgA titres in lung (Figure 2A; p < 0.001) compared to negative control lambs but no additive effect was observed compared to parenteral control lambs. Finally, although the anti-OVA IgA response in respiratory mucosa generated by lambs gavaged with 0.23 g OVA for 3 days was significant relative to the negative control group (Group B; p < 0.05), the response was much less robust compared to the parenteral control group. Next we assessed the titres of anti-OVA IgG in the respiratory mucosa with and without prior oral exposure to OVA. We observed that lambs gavaged with 0.23 g OVA for 3 days (Group B), 0.023 OVA for 9 days (Group C) and the parenteral control group generated a trend towards increased anti-OVA IgG titres in lung washes compared to control lambs although the data did not meet the criteria set for statistical significance (Figure 2B, p < 0.065). Lambs gavaged with a single bolus of 2.27 g OVA prior to i.p. immunization showed negligible titres of anti-OVA IgG in the lung lavage which may indicate induction of oral tolerance. Collectively, these results suggest that prior oral antigen exposure in newborn lambs may impact the magnitude of response to i.p. immunization.

### Gavage of neonatal lambs with OVA did not further promote induction of OVA-specific cell-mediated immunity

**Figure 3:**
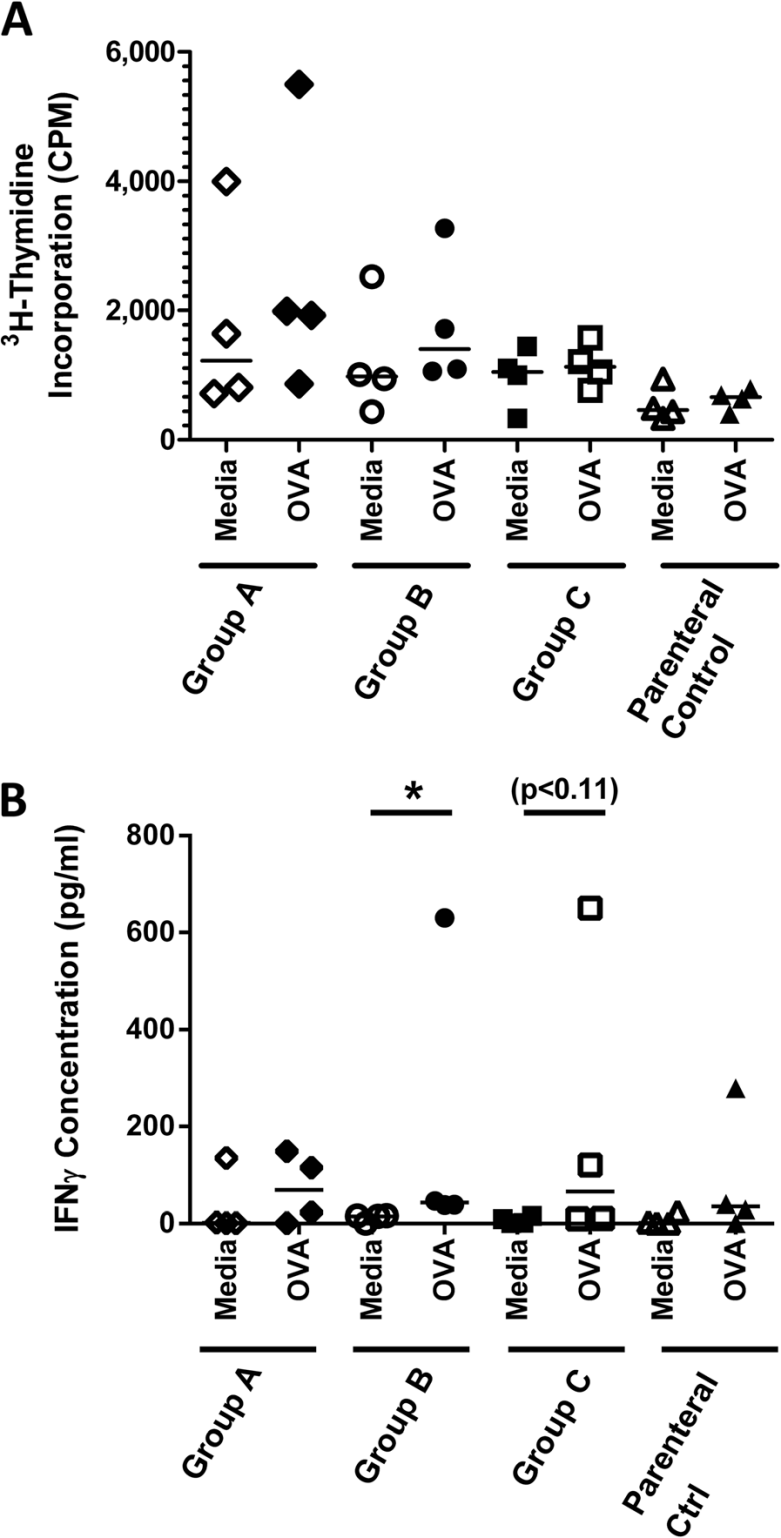
**OVA-specific cytokine production by splenocytes from lambs gavaged with OVA then i.p. immunized with OVA at 4 weeks of age.** Lambs (n = 4/group) were gavaged and i.p. immunized as described in Figure 1A. Lymphocyte proliferation **(A)** and IFN-γ **(B)** production from ex *vivo* re-stimulated splenocytes was measured by ELISA 3 weeks post i.p. immunization. Each data point represents an individual animal and median values are indicated by horizontal lines. *p < 0.05.

Finally, we sought to determine whether lambs orally gavaged with OVA starting the day after birth developed cell-mediated immunity. Spleens were excised from each lamb at 7 weeks of age and splenocytes were restimulated with OVA or media. Splenocytes did not show OVA-specific induction of lymphocyte proliferation or interleukin (IL)-4 cytokine production in any groups (Figure 3A and data not shown). However, splenocytes from lambs gavaged with 0.23 g OVA over 3 days (Group B) generated significant antigen-specific IFN-γ production compared to unstimulated cells (Figure 3B, p < 0.05). Splenocytes from lambs in Group C (p < 0.11) also showed a trend towards OVA-specific IFN-γ production but this data was not statistically significant. From the lambs in Group A and the parenteral control lambs, two had splenocytes which produced IFN-γ in response to *ex vivo* antigen-exposure. It is possible that lambs exposed for the shorter period with the higher dose (Group A) experienced limited induction of cellular immunity but not a humoral response. More animals will be needed to establish whether this is indeed the case. These data indicate that oral gavage of newborn lambs did not have any additive effect over what was observed in the group immunized via the i.p. route alone.

## Discussion

The present investigation showed that both systemic and mucosal humoral immune responses were induced following oral immunization of conventionally reared newborn lambs repeatedly exposed to 0.023 g OVA and primed with OVA in IFA by the i.p. route. Traditionally, immunization of the very young has been avoided because it was presumed that the neonatal immune system was too immature to respond. However, GALT in ruminants displays extensive fetal and neonatal development and indeed responsiveness to infectious agents [23]. Oral inoculation of foals with virulent *Rhodococcus equi* bacteria demonstrated accelerated cytotoxic T lymphocyte development and IFN-γ production [24]. In sheep, Emery et al. determined that lambs repeatedly infected with infectious larvae of *Haemonchus controtus* or *Trichostronglyus colubrifomis* starting from the day of birth for 4–6 weeks showed significant reduction in mean faecal egg count compared to control lambs [25,26]. Importantly, the cell-mediated immune responses (i.e. antigen-specific cellular proliferative response and IFNγ production) by trickle-immunised lambs was not significantly greater than that in control animals, which corroborates our CMI response [25]. Although at least 50% of all vaccinated animals some induction of IFNγ compared to media controls, the level of response was very low (Figure 3A) and no proliferative response was observed (Figure 3B). Emery et al. also show that lambs were protected despite no significant increase in serum antibody production [25].

When they repeated their trial to include lambs repeatedly infected with infectious *T. colubrifomi* larvae, only animals also immunised i.p. with 50 μg of the recombinant *T. colubriformis* derived protein in the presence of IFA showed induction of IgG_1_ and IgG_2_ isotypes antibody secreting cells in mesenteric lymph nodes [26]. In contrast, data from our study showed significant induction of OVA-specific serum IgG prior to and post i.p. immunization (Figure 1A-C) and significant mucosal IgA but not IgG was induced after i.p. immunization (Figure 2A, B). Experiments by Mutwiri et al. (2001) determined that consistent exposure of a localized region of the newborn GALT to antigen was sufficient to promote mucosal and systemic immunity [15]. Specifically, they localized adenovirus coding for TgD antigen to a segment of the newborn gut. They presumed that antigen would be consistently expressed (although the levels and duration of expression were not assessed [27]). While their results were intriguing, antigen in this study was introduced to the gut with several potentially confounding factors. Because the immunized intestinal ‘loops’ were created in fetal lambs at late gestation and the ‘loops’ were made to be sterile, the antigen was not diluted out by the presence of commensal flora. Further, because the ‘loops’ were removed from the active digestive tract (while still keeping lymphatics, enervation and blood flow intact), antigen was not influenced by the potentially inhibitory factors present in colostrum and milk and the peristaltic action of the gut. Regardless, these experiments showed that antigen alone (or at least one coded for by adenovirus) introduced to a localized region of a sterile gut could promote mucosal and systemic immunity in newborn lambs. Our data expanded upon this research by establishing that oral antigen alone could promote immunity in conventionally reared newborn lambs despite being colonized with commensal bacteria. Specifically, lambs gavaged with 0.023 g OVA persistently for 9 days showed induction of anti-OVA IgG in serum after 4 weeks even without localizing the antigen to a specific region of the gut. If oral tolerance to OVA had been induced, re-exposure to the antigen by intraperitoneal injection should have resulted in a reduction of serum anti-OVA IgG titres after 7 weeks. Instead, we observed that the anti-OVA IgG titres increased relative to what was observed after oral exposure alone (week 4). In fact, average serum titres from lambs gavaged with 0.023 g OVA for 9 days (Average = 2.3 × 10^∧ 5^+/−1.7 × 10^∧ 5^ (StDev) were 1.8 fold higher than was what observed in lambs injected with OVA i.p. without prior oral exposure (Parenteral control group; Average = 1.3 × 10^∧ 5^+/−1.2 × 10^∧ 5^ (StDev)). Therefore, persistent low dose antigen exposure by the oral route can promote immunity in lambs. However, our experimental design is limited in that it is unclear whether the low dose or duration of exposure (or both) contributed to the immune response. Further studies must be undertaken to clarify the precise dose and duration of exposure required for induction of immunity as well as including direct measurements of mucosal immunity. This knowledge will contribute to our understanding of the underlying mechanisms by which this vaccination strategy promotes immunity.

Immunization in the perinatal period should be amenable to current animal husbandry practices as large animals are often segregated from the herd prior to lambing/calving/farrowing and are accessible to producers. Oral gavage (drenching) is routinely performed by producers without the need for veterinary assistance and oral administration negates the need for needles which reduces risk of carcass condemnation in meat-producing animals. However, we are aware that repeated oral drenching over a period of days is not likely going to be embraced by producers unless the results are robust enough to warrant this approach, which is not the case with our data. We submit that the results are intriguing enough to warrant further study. Future experiments should include larger numbers of lambs per group to establish how this vaccination strategy affects an out-bred animal population. We intend to study whether a single (or at most two) oral gavage(s) are sufficient to promote immunity if the dose is low (i.e. 0.023 g OVA on the day of birth and/or for 2 days after birth) which is much more likely to be accepted by the livestock industry should the results prove favourable. It is known that the gut of newborn ruminants is semi-permeable for only the first 24–36 hours after birth to facilitate passive transfer of maternal antibodies from the colostrum [28]. Dendritic cells (DCs) within the sub-epithelial dome may present antigens to T cells in the Peyer’s Patch or isolated lymphoid follicle (ILF) [29-31] rather than being taken up by tolerogenic mucosal DCs which sample the intestinal lumen then migrate preferentially to the mesenteric lymph nodes (MLNs) upon antigen uptake [32]. If induction of oral immunity requires that the antigen traverse the gut wall, it may be that despite administering OVA for 9 days to lambs in Group C, only the first 2–3 doses traversed the gut wall and led to induction of oral immunity. Experiments are underway to elucidate the kinetics of gut permeability and the impact this has on the mechanisms of antigen uptake and where antigen presentation to lymphocytes occurs (i.e. in the Peyer’s patches, isolated lymphoid follicles, or mesenteric lymph nodes). Should future studies show that early life oral vaccination consistently promotes oral immunity, it will have important implications for protecting against infectious diseases in the very young and it may reduce the number of carriers of disease-producing organisms within a herd.

In contrast to the immune response induced in neonatal lambs gavaged with OVA over a 9 day period immediately after birth, a single high dose exposure to antigen appeared to promote a trend towards induction of oral tolerance in lambs. Our results suggest that inappropriate induction of oral tolerance to an antigen may impede the host from generating an immune response to this antigen in the future. These results corroborate what was observed by Buddle et al. (2002) wherein it was observed that already at 6 weeks of age, calves had been naturally exposed to mycobacterial antigens [33]. As adults, the cattle with pre-existing responses to environmental mycobacteria were much less responsive to vaccination than were naïve adult cattle [33]. Further experiments must be performed to clearly determine whether in fact, as our data appears to indicate, early-life antigen exposure in lambs also negatively impacts future response to vaccination. Should this prove to be the case, it will be critical to establish 1) whether a parenteral vaccine can be administered to circumvent or reverse the tolerogenic response, or 2) whether it is advisable to proactively orally vaccinate neonates prior to environmental exposure to preserve their ability to promote an immune response to later pathogen or vaccine exposure events.

## Conclusions

In the present study, we determined that repeated low dose oral administration of OVA for 9 days of age starting the day after birth induced priming of the immune system, not oral tolerance. In contrast, a single, higher dose oral exposure in neonatal lambs promoted what appeared to be a tolerogenic response which negatively impacted the immune response to parenteral vaccination in later life. We conclude that neonatal lambs are receptive to either induction of immunity or oral tolerance if exposed to antigen in the period immediately after birth, depending on the dose and/or kinetics of exposure. These results are intriguing and should be studied further to establish whether it may be advisable to proactively orally vaccinate newborn lambs not only to promote immunity but to ensure that the they can respond appropriately to parenteral vaccines in later life.

## Methods

### Immunization procedure

This work was approved by the University of Saskatchewan’s Animal Research Ethics Board, and adhered to the Canadian Council on Animal Care guidelines for humane animal use. Pregnant Suffolk-cross ewes were housed at VIDO for at least 3 days prior to lambing with *ad libitum* access to standard feed and water. Ewes were subjected to intramuscular injection with 3 ml of 5 mg/ml Dexamethasone (Dexocort-5, Rafter, Calgary, AB) at day −1 to promote labour. We gavaged lambs with a single bolus of 2.27 g OVA, 0.23 g OVA daily for 3 days, or 0.023 g OVA (Sigma-Aldrich Canada Ltd, Oakville, ON, Canada) for 3 consecutive days followed by oral gavage at the same dose at 5 days, 7 days and 9 days (Figure 1A). Lambs were randomly assigned to treatment groups. A total volume of 60 ml was administered via gavage using soft Nalgene Tubing with a monojet catheter tip (Fisher Scientific Ltd., Ottawa, ON) gently inserted into the back of the throat. Although not all lambs were born on the same day, the timing of gavages and other treatments were maintained for each according to that described in Figure 1A. At 4 weeks age, lambs were i.p.-injected with 10 mg OVA plus IFA (Sigma-Aldrich Canada Ltd.). To generate a parenteral control group, lambs received saline by oral gavage and they were i.p. immunization with OVA as indicated above. This route was used because it is considered relevant for stimulating the mucosal tissues [11,34]. Control lambs (see Figure 2) did not undergo any form of immunization. Sheep were euthanized using 2 ml/10 lb body weight with Pentobarbital Sodium Injection (240 mg/ml; Euthanyl, Bimeda-MTC Animal Health Inc., Cambridge, ON). Sera was obtained after 3 weeks, after 4 weeks (immediately prior to i.p. immunization) and at 7 weeks of age. Lung lavages and spleens were obtained at the end of the trial (7 Weeks; Figure 1A). Endotoxin levels in OVA was determined to be 8,000 U/ml using the Limulus Amebocyte Lysate enzymatic assay QCL-1000 (Lonza Group Ltd, Basel, Switzerland) according to the manufacturer’s instructions.

### Serum and lung lavages ELISA

To measure OVA-specific antibody titres, blood sera and lung lavages was obtained as previously described [35,36]. Immunolon II microtiter plates (Dynex Technology Inc., Chantilly, VA, USA) were coated overnight at 4°C with OVA at 10 μg/ml in carbonate coating buffer (15 mM Na_2_CO_3_, 35 mM NaHCO_3_, pH 9.6) and 100 μl of antigen was added to each well. Wells were washed 5 times with Tris-buffered saline (pH 7.3) containing 0.05% Tween 20 (TBST). Diluted sheep serum samples were added to the wells at 100 μl/well and incubated overnight at 4°C. Wells were washed again with TBST and biotinylated rabbit anti-sheep IgG (KPL, Gaithersburg, MD, USA, #16-23-06, 1/5000), or rabbit anti-sheep IgA (Bethyl Laboratories, Montgomery, TX, USA; 1/1,000) (which was biotinylated in-house using standard techniques) were added to the wells in a 100 μl volume and incubated for 1 h at room temperature (RT) where appropriate. Wells were washed alkaline phosphatase conjugated with streptavidin (Jackson Laboratories, CA, USA; 1/5,000) was added to each well followed by incubation for 1 h at RT. Wells were washed 5 times in TBST before di(Tris) p-nitrophenyl phosphate (PNPP) (Sigma-Aldrich Canada Ltd.) was diluted 1/100 in PNPP substrate buffer (1 mM of MgCl_2_, 200 mM of Tris–HCl, pH 9.8) and 100 μl/well was added to the wells. The reaction was allowed to develop for 15 min before absorbance was read as optical density (OD) at 405 nm in a Microplate Reader (Bio-Rad Laboratories, CA, USA). Results were reported as titres which are the reciprocal of the highest dilution that gave a positive OD reading. A positive titre was defined as an OD reading that was at least two times greater than the values for a negative sample. All ewes showed negligible anti-OVA IgG in their serum and colostrum (Figure 1B-D).

### Splenocyte cytokine ELISAs

To measure systemic immune responses, splenocytes were isolated, processed, and cultured in triplicate wells at a concentration of 6 × 10^5^ cells/well as detailed in [35] in the presence of 10 μg/mL OVA or media for 18–20 h. Ovine IFN-γ ELISA kit (MABTECH, Mariemont, OH, USA) were performed according to manufacturer’s recommendations. For ovine IL-4, polystyrene microtitre plates were coated with bovine IL-4 (AbD Serotech, Raleigh, NC, USA; MCA1820) used at 1 μg/ml in 100 μl volume with carbonate coating buffer and incubated at 4°C overnight as detailed above. Wells were washed 5 times with Tris buffered saline (pH 7.3) containing 0.05% Tween 20 (TBST). Diluted sheep supernatants were added to the wells at 100 μl/well and incubated for 2 h RT. Wells were washed again with TBST and bound cytokines were detected using 1 μg/ml in 100 μl mouse anti-bovine antibody (sc-101845, Santa Cruz Biotech, Santa Cruz, CA, USA.) which was introduced for 1 h at RT. Wells were washed and alkaline phosphatase conjugated goat anti-mouse IgG (KPL, 1/5000) was added to each well followed by 1 h incubation at RT. Wells were washed 5 times in TBST and detected as detailed above. Serially diluted recombinant bovine IL-4 was used as standards. For each cytokine ELISA, standard curves were constructed to calculate the cytokine concentrations in the samples. Values were corrected for background cytokine secretion by subtracting the concentrations in the control (medium) wells.

### Lymphocyte proliferative response assays

Following the procedure outlined in [37], spleens were isolated, processed, and cultured in 96-well flat-bottom plates (Nalge Nunc International, Naperville, IL, USA) at 3 × 10^5^ cells/well in a final volume of 200 μl culture medium with triplicate wells containing either medium alone or 10 μg/ml OVA. Cells were incubated for 72 h followed by the addition of 0.4 μCi ^3^H-thymidine (Amersham Pharmacia Biotech, Baie d’Urfe QC, Canada)/well for another 16 h of culture. Cells were freeze-thawed and harvested onto Unifilter plates (Perkin-Elmer, Boston, MA, USA) and incorporation of ^3^H-thymidine was measured as counts per minute (cpm) using a liquid scintillation counter (Top-Count, Perkin-Elmer).

### Statistical analysis

The outcome data from this study were not normally distributed. Therefore, differences among experimental groups were tested using Kruskal-Wallis analysis and medians were compared using Dunn’s test. In those instances where repeated measures were made on the same animals over time, the data for each animal were summed over the duration of the study and then Kruskal-Wallis analysis and Dunn’s test were performed on the sums. Differences were considered significant if P < 0.05. All statistical analyses and graphing were formed using GraphPad Prism 5 software (GraphPad Software, San Diego, CA).

## Abbreviations

BCG: Bacillus calmette-guérin; BALs: Broncheoalveolar lavage; CMI: Cell-mediated immune response; Cpm: Counts per minute; DCs: Dendritic cells; Tris: Di; PNPP: P-nitrophenyl phosphate; ELISA: Enzyme-linked immunosorbent assays; GALT: Gut-associated lymphoid tissues; H: Hour; IL: Interleukin; IFA: Incomplete freund’s adjuvant; i.p.: Intraperitoneal; IFLs: Isolated lymphoid follicles; MW: Molecular weight; M. bovis: *Mycobacterium bovis*; OVA: Ovalbumin; OD: Optical density; PFU: Plaque forming units; PPD: Purified protein derivative; RT: Room temperature; StDev: Standard deviation; TBST: Tris-buffered saline with tween 20; VIDO: Vaccine & infectious disease organization.

## Competing interests

The authors declare that they have no competing interests.

## Authors' contributions

HLW conceived of and designed the experiments and wrote the manuscript. RMB and SM performed the laboratory experiments. All authors read and approved the final manuscript.
